# Supplementary material for: Tissue-based IL-10 signalling in helminth infection limits IFNγ expression and promotes the intestinal Th2 response
Source: Mucosal Immunol. 2022 Apr 15;15(6):1257–69. doi: 10.1038/s41385-022-00513-y (PMC9705258; doi:10.1038/s41385-022-00513-y)
Supplement: Supplementary file 1 — Supplementary Data [file 41385_2022_513_MOESM1_ESM.pdf]

Supplementary Data

Tissue-based IL-10 signalling in helminth infection limits IFNγ expression and promotes the intestinal Th2 response

Holly C. Webster, Virginia Gamino, Anna T. Andrusaite, Olivia Ridgewell, Jack McCowan, Amy L. Shergold, Graham A. Heieis, Simon W. F. Milling, Rick M. Maizels, Georgia Perona-Wright

Figures S1-S7

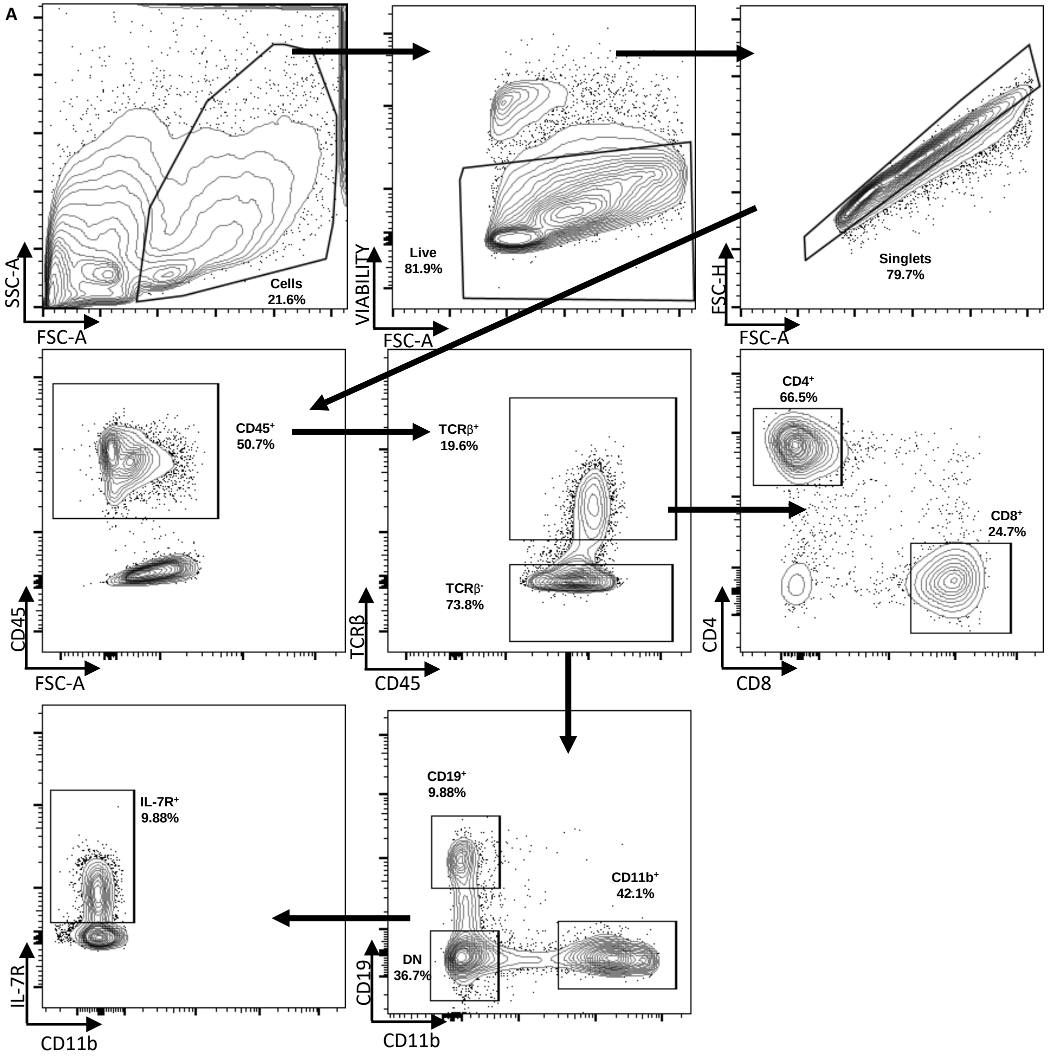

Supplementary Figure 1. Gating for cell subsets in the small intestine during *H. polygyrus* infection

Il10gfp-foxp3rfp B6 mice were infected with 200 L3 *H. polygyrus* and 7 days later the small intestine and MLN collected for analysis. Representative gating of cell subsets from D7 *H. polygyrus* infected small intestine.

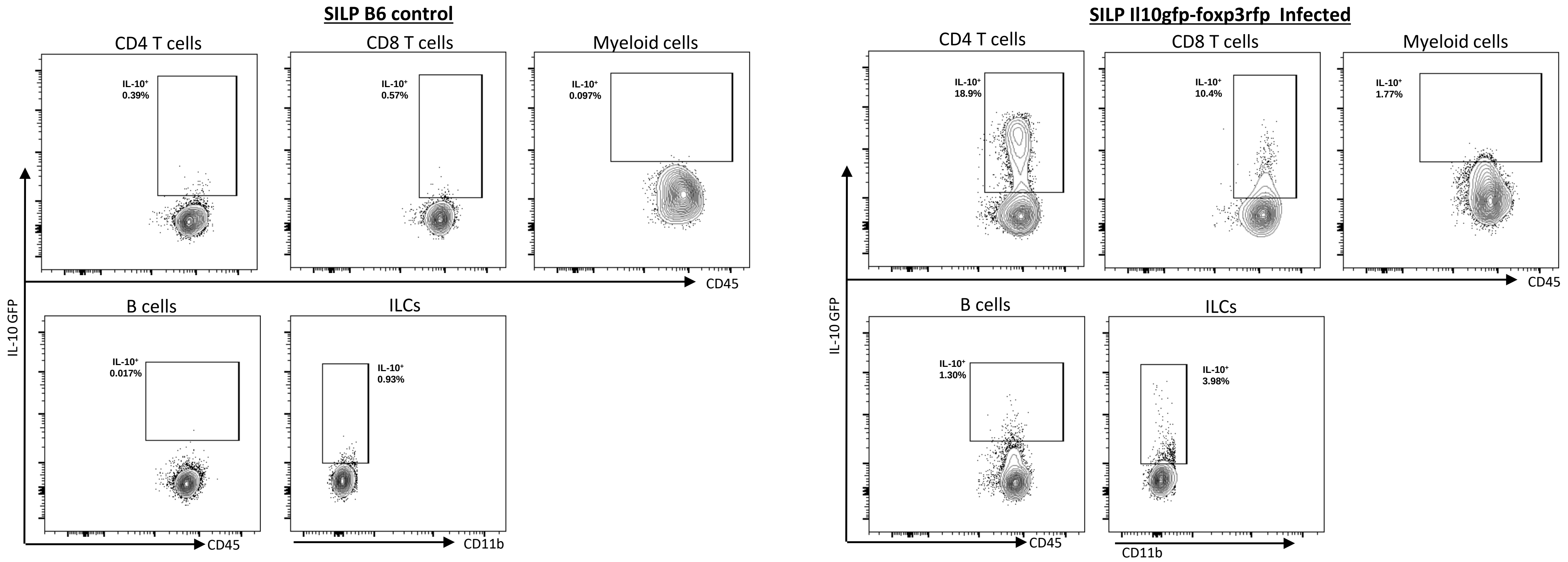

Supplementary Figure 2. IL-10 gating for cell subsets

Il10gfp-foxp3rfp OR C57BL/6 mice were infected with 200 L3 *H. polygyrus* and 7 days later the small intestine and MLN collected for analysis. Representative gating for IL-10 from each cell subset from D7 *H. polygyrus* infected small intestine and B6 control mice. All gates were drawn using B6 control mouse as a negative control.

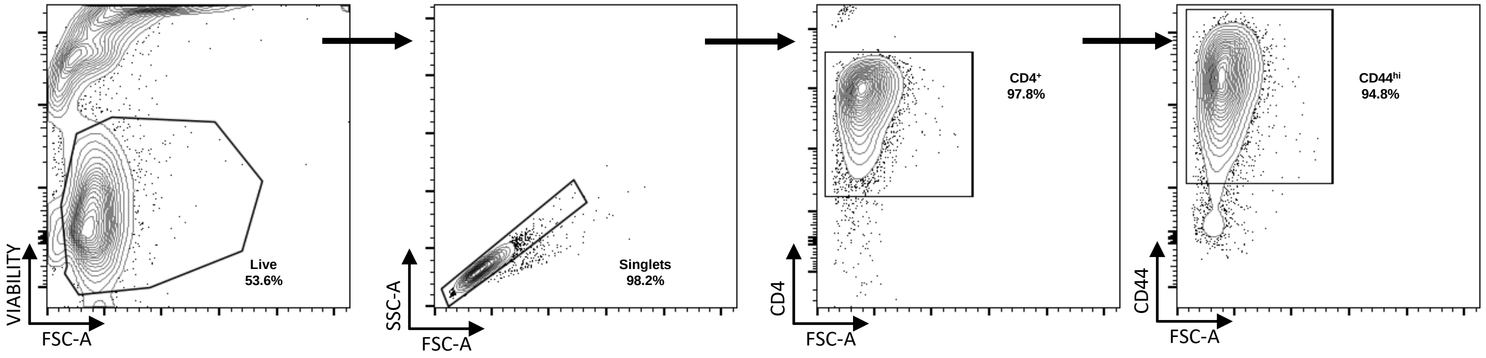

Supplementary Figure 3. ***In vitro*** **CD4 T cell gating**

In vitro polarised Th0 cells were cultured with αCD3, αCD28 and IL-2 for 4 days and harvested for further analysis. Representative gating strategy of *in vitro* activated Th0 cells.

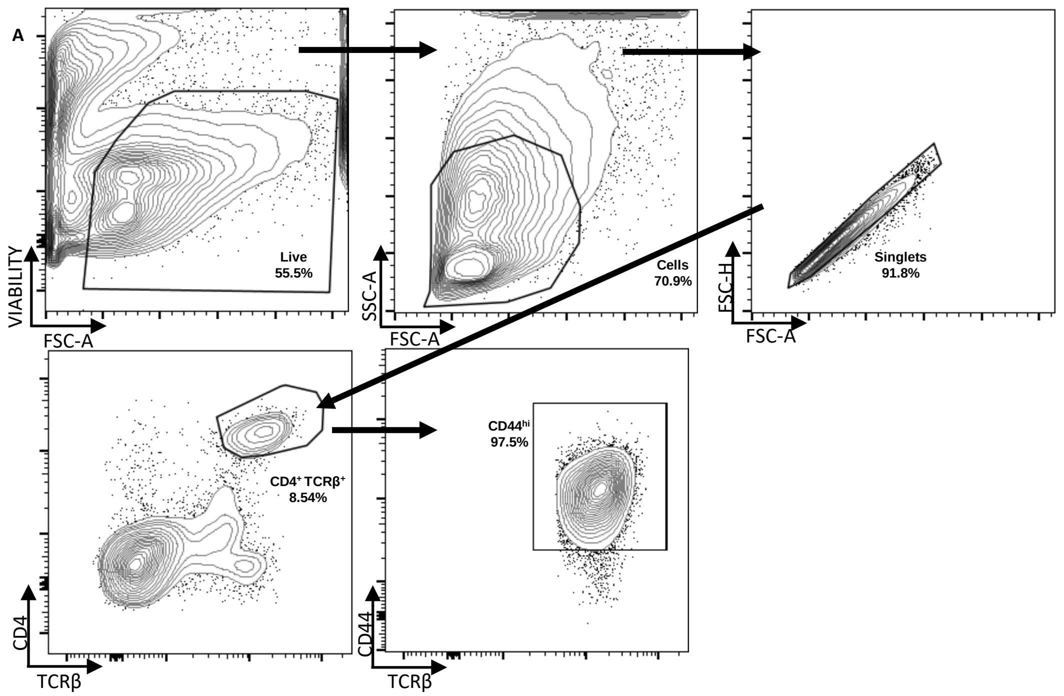

Supplementary Figure 4. *In vivo* CD4 T cell gating
C57BL/6 mice were infected with 200 L3 *H. polygyrus* and at D-1, D2 and D5 treated with anti-IL-10R mAb or isotype control, and 7 days post-infection the small intestine and MLN collected for analysis. Representative gating of activated CD4 T cells from D7 *H. polygyrus* infected small intestine.

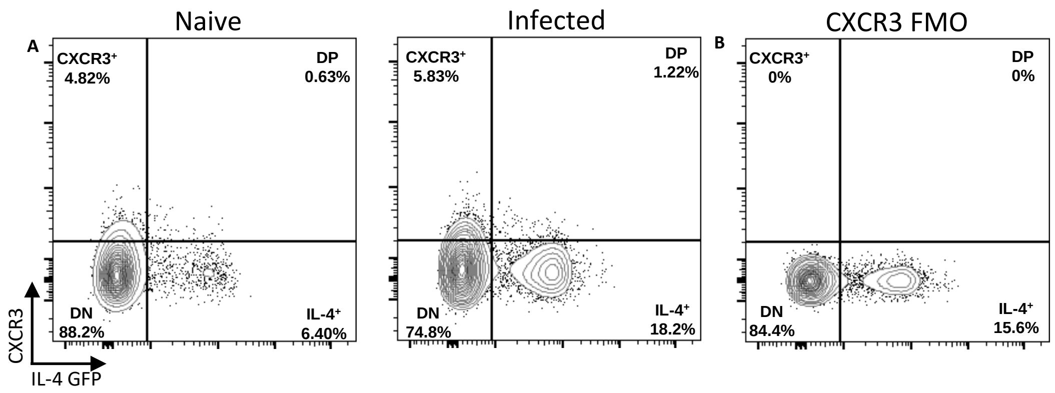

Supplementary Figure 5. IL-4 and CXCR3 staining

B6 4get mice were infected with 200 L3 *H. polygyrus* and 7 days post-infection the small intestine and MLN removed. (A) Representative staining for IL-4 and CXCR3 expression by CD4 T cells from D7 *H. polygyrus* naïve and infected small intestine. (B) Representative staining from CXCR3 FMO control used to gate on CXCR3 positive cells.

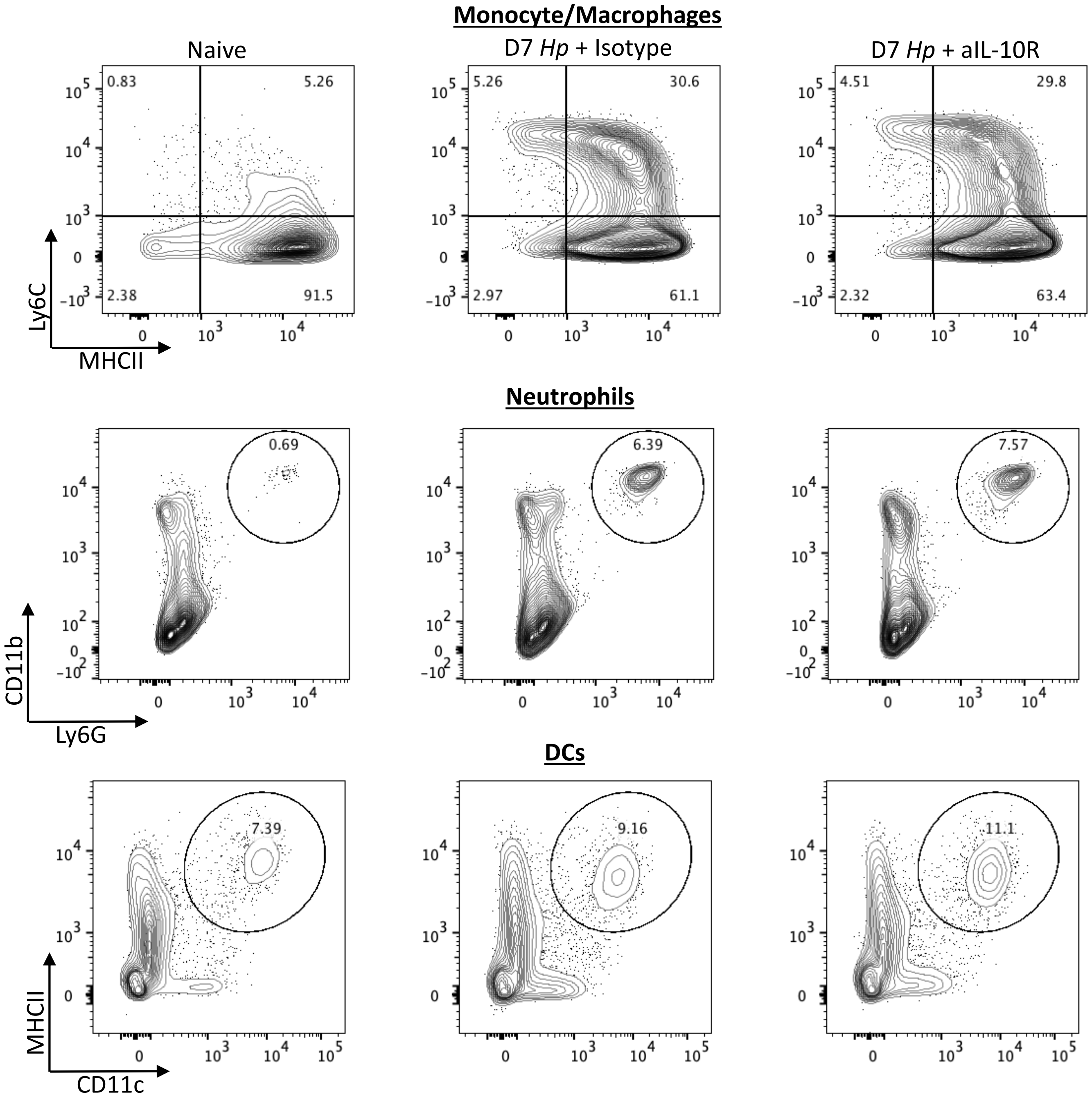

Supplementary Figure 6.
***In vivo* myeloid cell gating**
C57BL/6 mice were infected with 200 L3 *H. polygyrus* and at D-1, D2 and D5 treated with anti-IL-10R mAb or isotype control, and 7 days post-infection the small intestine collected for analysis. Representative gating of myeloid cell subsets from D7 *H. polygyrus* infected SILP.

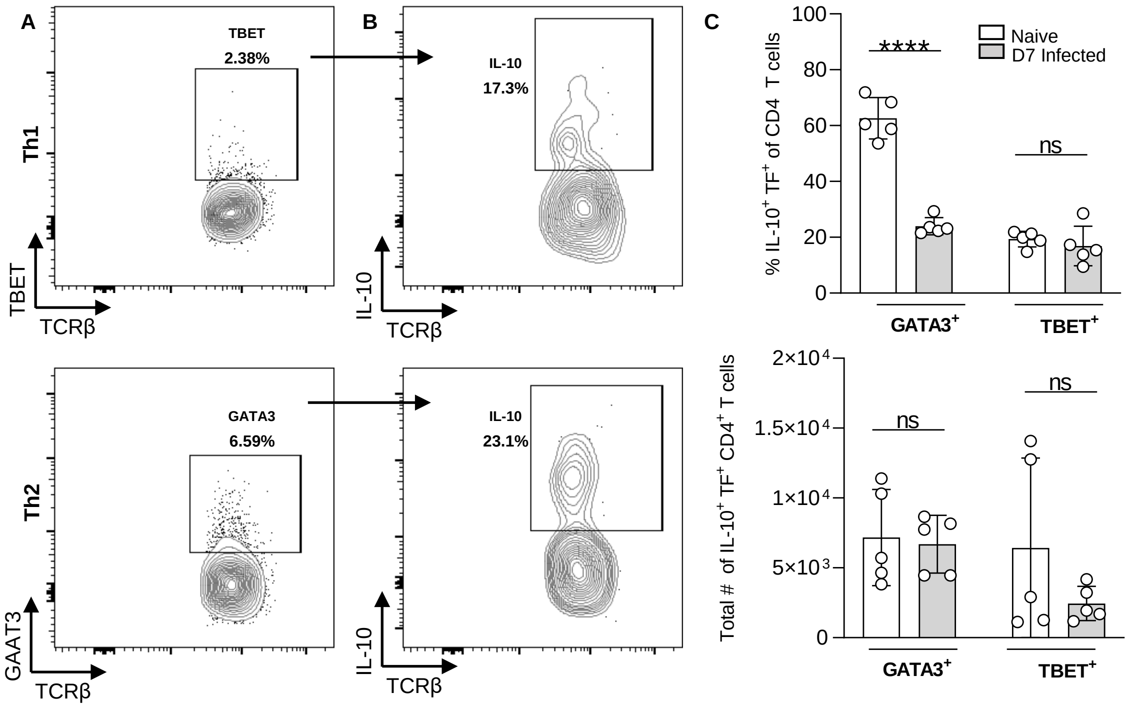

Supplementary Figure 7. IL-10 expression by Th1 and Th2 cells during H. polygyrus infection

Il10gfp-foxp3rfp B6 mice were infected with 200 L3 *H. polygyrus* and 7 days later the small intestine collected for analysis. (A) Representative flow cytometry plot of TBET (top) and GATA3 (bottom) staining from the SILP of D7 Hp infected mice. (B) Representative flow cytometry plot of IL-10$^+$ (anti-GFP) from (top) TBET$^+$ and (bottom) GATA3 (bottom) CD4 T cells from the SILP of D7 Hp infected mice. (C) Percentage of (top) and total number of (bottom) IL-10$^+$ (anti-GFP) from GATA3$^+$and TBET$^+$ cells in the SILP. Graphed data are shown with means ± 1 SD and are representative of 1 independent experiment with n=5. Statistical significance was calculated by Student *t* test (Significance ****p< .0001).
