# Supplementary material for: Tissue-based IL-10 signalling in helminth infection limits IFNγ expression and promotes the intestinal Th2 response
Source: Mucosal Immunol. 2022 Apr 15;15(6):1257–69. doi: 10.1038/s41385-022-00513-y (PMC9705258; doi:10.1038/s41385-022-00513-y)
Supplement: Supplementary file 2 — Author checklist [file 41385_2022_513_MOESM2_ESM.pdf]

NOTE: Please save this file locally before filling in the table, DO NOT work on the file within your internet browser as changes will not be saved. Adobe Acrobat Reader (available free here) is recommended for completion.

The ARRIVE guidelines 2.0: author checklist

The ARRIVE Essential 10

These items are the basic minimum to include in a manuscript. Without this information, readers and reviewers cannot assess the reliability of the findings.

Item		Recommendation	Section/line number, or reason for not reporting
**Study design**	1	For each experiment, provide brief details of study design including:	
		a. The groups being compared, including control groups. If no control group has been used, the rationale should be stated.	Provided in main text and in figure legends
		b. The experimental unit (e.g. a single animal, litter, or cage of animals).	Stated in figure legends (normally a single animal)
**Sample size**	2	a. Specify the exact number of experimental units allocated to each group, and the total number in each experiment. Also indicate the total number of animals used.	Stated in figure legends
		b. Explain how the sample size was decided. Provide details of any *a priori* sample size calculation, if done.	Power calculations done prior to start of project, using previous studies to estimate expected differences
**Inclusion and exclusion criteria**	3	a. Describe any criteria used for including and excluding animals (or experimental units) during the experiment, and data points during the analysis. Specify if these criteria were established *a priori*. If no criteria were set, state this explicitly.	No animals were excluded during this study. Exclusion criteria are animal death unrelated to this study, and/or a technical fail during sample processing.
		b. For each experimental group, report any animals, experimental units or data points not included in the analysis and explain why. If there were no exclusions, state so.	No exclusions were made
		c. For each analysis, report the exact value of *n* in each experimental group.	Stated in each figure legend
**Randomisation**	4	a. State whether randomisation was used to allocate experimental units to control and treatment groups. If done, provide the method used to generate the randomisation sequence.	Mice were considered identical before the start of the study, and were allocated in turn to each experimental group
		b. Describe the strategy used to minimise potential confounders such as the order of treatments and measurements, or animal/cage location. If confounders were not controlled, state this explicitly.	Mice treated with aIL-10R or isotype control were co-housed in the same cages, to remove cage confounders
**Blinding**	5	Describe who was aware of the group allocation at the different stages of the experiment (during the allocation, the conduct of the experiment, the outcome assessment, and the data analysis).	Blinding was only used during the scoring of our histology slides
**Outcome measures**	6	a. Clearly define all outcome measures assessed (e.g. cell death, molecular markers, or behavioural changes).	Outcome measures are described in the main text and in each figure legend
		b. For hypothesis-testing studies, specify the primary outcome measure, i.e. the outcome measure that was used to determine the sample size.	The primary outcome for each experiment is explained in the main text
**Statistical methods**	7	a. Provide details of the statistical methods used for each analysis, including software used.	Statistical analyses are described in each figure legend, and in a paragraph in the Methods section
		b. Describe any methods used to assess whether the data met the assumptions of the statistical approach, and what was done if the assumptions were not met.	Data was tested for normal distribution, and appropriate statistical tests were then applied
**Experimental animals**	8	a. Provide species-appropriate details of the animals used, including species, strain and substrain, sex, age or developmental stage, and, if relevant, weight.	Included in the first paragraph of the Methods section
		b. Provide further relevant information on the provenance of animals, health/immune status, genetic modification status, genotype, and any previous procedures.	Included in the first paragraph of the Methods section, plus cited references to the original publications of the individual mouse strains
**Experimental procedures**	9	For each experimental group, including controls, describe the procedures in enough detail to allow others to replicate them, including:	Included in the Methods section, for each procedure. The rationale is provided in the main text.
		a. What was done, how it was done and what was used.	
		b. When and how often.	
		c. Where (including detail of any acclimatisation periods).	
		d. Why (provide rationale for procedures).	
**Results**	10	For each experiment conducted, including independent replications, report:	Each graph depicts the mean plus/minus the standard deviation
		a. Summary/descriptive statistics for each experimental group, with a measure of variability where applicable (e.g. mean and SD, or median and range).	
		b. If applicable, the effect size with a confidence interval.	
